# Comparing Wafi ileostomy to Brooke ileostomy in stage 3 rectal cancer: a prospective cohort study

**DOI:** 10.1007/s13304-025-02305-y

**Published:** 2025-06-24

**Authors:** W. Attallaah, M. Gachabayov, A. Bulut, O. Verdiyev, M. Javadov, E. Barzola, O. Inanc, A. Kajmolli, R. Bergamaschi

**Affiliations:** 1 Department of Surgery, Marmara University, Istanbul, Turkey; 2 Section of Colorectal Surgery, Westchester Medical Center, New York Medical College, Valhalla, NY USA; 3 Jacobi Medical Center, Suite 610, 1400 Pelham Parkway South, New York, NY 10461 USA

**Keywords:** Rectal cancer, Ileostomy, Ileostomy reversal, Tube ileostomy, Loop ileostomy

## Abstract

The aim of this study was to compare Wafi ileostomy to Brooke ileostomy in patients undergoing elective resection for stage 3 rectal cancer in terms of ileostomy creation and reversal-related complications. This was a prospective cohort study enrolling consecutive patients with stage 3 distal rectal cancer undergoing elective TME with Wafi or Brooke ileostomy at a median 8-week interval following neoadjuvant chemoradiation in two institutions. Wafi ileostomy was defined as the insertion of a soft polyvinylchloride spiral endotracheal tube into the afferent limb of the terminal ileum with a flexible rubber band passed behind the backwall of its efferent limb to occlude. Brooke ileostomy was defined as the exteriorization of the terminal ileum afferent limb through the abdominal wall (then everted and sutured to the skin) with the efferent limb acting as mucous fistula. Propensity score matching with a 1:1 ratio was employed to compare diagnosis-matched patients for age, gender, and American Society of Anesthesiologists score. During 5 years, 110 patients underwent TME with Wafi ileostomy, whereas 116 patients underwent TME with Brooke ileostomy. Propensity score matching left 99 Wafi and 99 Brooke comparable patients. Wafi ileostomy was reversed (tube removed at bedside) at median postoperative day (POD) 14 (same hospital stay) as compared to 150 days of ileostomy reversal (second surgery) in the Brooke ileostomy group (*p* < 0.001). Ileostomy-related overall complication rates were significantly lower in Wafi ileostomy patients (6% vs. 24%, *p* = 0.001). On multivariable logistic regression, dehydration was found to be associated with increased emergency room visits and readmissions in Brooke ileostomy patients (OR = 1.24 (1.03, 3.92); *p* = 0.044). Compared to Brooke, Wafi ileostomy with its reversal at bedside without need for a second surgery was associated with fewer complications.

## Introduction

Anastomotic leakage (AL) remains a critical complication of rectal cancer surgery, leading to increased re-operation rates, extended hospital stays, postponed adjuvant oncologic therapy, and higher mortality rates [1]. Although the rate of AL has been recently reported to be 4.6% in a retrospective cohort study by the US Rectal Cancer Consortium [2], the said rate has been previously found to range from 10 to 20% depending on the duration of follow-up [1]. Recent data from a literature review [3] and a population-based study [4] have both reported AL rates ranging from 3 to 8%, whereof 75% occurring at the rectal anastomosis and resulting in mortality rates ranging from 1.7 to 16.4% [1, 2].

Prior to Brooke’s undramatic 1952 description of a procedure “to evaginate the ileal end and suture the mucosa to the skin” [5], an ileostomy was a traumatic event for patients to be confronted with. In fact, the ileum was brought out several inches with a tube inserted [6], and it was later skin grafted in an attempt to protect the abdominal wall and pass the corrosive succus into the precursor of a today’s appliance [7]. Nowadays, it is known that although a loop ileostomy may alleviate some of the serious complications of AL (such as fecal peritonitis and septicemia) [2], it does not prevent AL per se [8]. Furthermore, an ileostomy may be associated with serious morbidity, ranging from physiologic complications [9] (including but not limited to dehydration, electrolyte imbalances, acute kidney failure) to local complications [10], as well as psychological distress. Moreover, a National Surgical Quality Improvement Program database study has reported that the reversal of a loop ileostomy can be associated with a 18% complication rate as well as a 0.6% mortality rate [11].

The search for alternatives to loop ileostomy may have started back in the 1970 s with transcaecal tube ileal diversion [12], a procedure that has been advocated for in the past decade [13], when a reemergence of interest for de-functioning colorectal anastomoses with a tube or a cannula appeared in the literature [14, 15]. Although the concept of tube ileostomy is not new, its adoption in clinical practice has been limited by concerns regarding its incomplete fecal diversion [16]. It is this limitation that prompted a study, which suggested applying a linear stapler approximately 10 cm distal to the tube ileostomy in an attempt to achieve complete diversion of the intestinal content [17]. However, in light of the fact that the staple line spontaneously recanalized during the postoperative period [17], Attaallah et al. accomplished complete diversion in 50 consecutive patients by applying a flexible rubber strip (to be tied over the skin) distal to the tube ileostomy [18]. Moreover, the latter authors introduced an additional modification by using a spiral endotracheal tube to prevent kinking and thereby occlusion of the tube.

The aim of this prospective cohort study enrolling consecutive patients with stage 3 distal rectal cancer undergoing elective total mesorectal excision following neoadjuvant therapy in two institutions was to compare tube to loop ileostomy in terms of ileostomy creation and reversal-related complications.

## Methods

### Study design, eligibility criteria, and end points

This was a prospective cohort study enrolling consecutive adult patients with stage 3 low rectal cancer undergoing elective total mesorectal excision with Wafi or Brooke ileostomy at a median 8-week interval following neoadjuvant chemoradiation in two institutions. The study was conducted in two centers between 2018 and 2022. All patients enrolled at Marmara University underwent Wafi ileostomy by one surgeon, whereas all patients enrolled at Westchester Medical Center underwent Brooke ileostomy by one surgeon. Ethical board approval was obtained at both institutions. Patients meeting one of the following criteria were not included in this study: (1) complications of rectal cancer (complete obstruction, bleeding, perforation) requiring emergent/urgent TME, (2) loss to follow-up after neoadjuvant chemoradiation, (3) history of small bowel disease requiring previous small bowel resection, (4) history of complex abdominal wall reconstruction, and (5) inability to obtain consent. The end points included rectal resection related complications, ileostomy creation related complications, ostomy output, length of stay, and complications of ileostomy reversal. Total length of stay was defined as length of stay from index rectal cancer surgery to discharge with reversed ileostomy.

### Study interventions

**Fig. 1:**
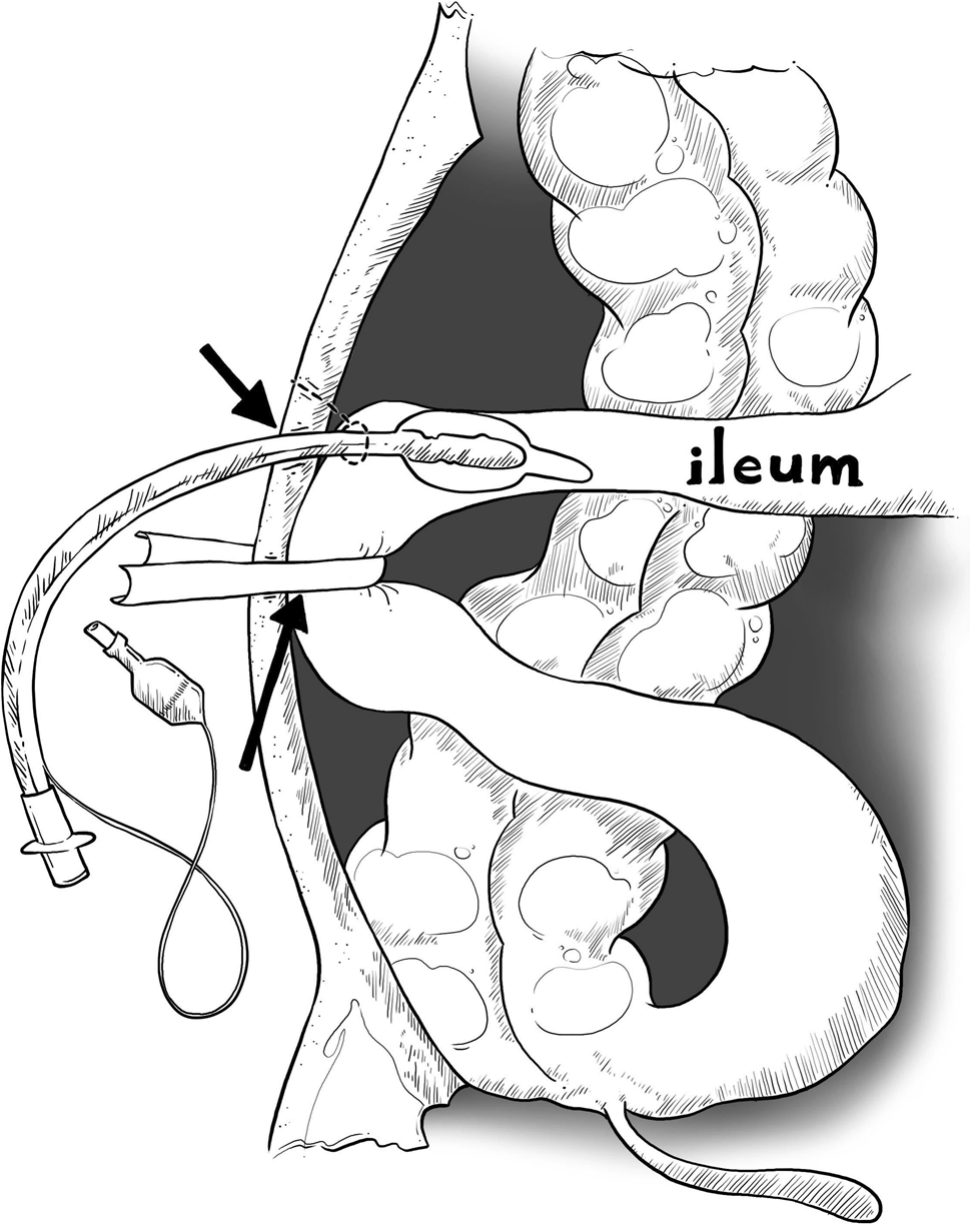
Wafi ileostomy

Wafi ileostomy was defined as the insertion of a soft polyvinylchloride 7.5 mm spiral endotracheal tube into the afferent limb of the terminal ileum with a flexible rubber band, namely a ¼ inch Penrose drain (Nantong Angel Medical Instruments Co. Ltd.; Nantong, China), which was passed behind the backwall of its efferent limb to occlude (Fig. 1). Reversal of Wafi ileostomy consisted of removal of the Penrose drain and the endotracheal tube at bedside, without any need for anesthesia or operating room. Brooke ileostomy was defined as the exteriorization of the terminal ileum afferent limb through the abdominal wall (then everted and sutured to the skin) with the efferent limb acting as mucous fistula. Reversal of Brooke ileostomy was a surgical procedure under general anesthesia, which entailed excision of the ileostomy site with small bowel resection and primary anastomosis.

### Statistical analysis

Propensity score matching with a 1:1 ratio was employed to compare diagnosis-matched patients for age, gender, and American Society of Anesthesiologists score. Median and interquartile range was the descriptive statistics used to express continuous variables, whereas categorical variables were expressed in numbers and percentage. Statistical analysis was performed using SPSS software (version 28.0; SPSS Inc., Chicago, IL, United States). Normality tests (Kolmogorov–Smirnov and Shapiro–Wilk tests) and histograms were used to test for normality of data distribution. Student’s *t*-test was used to compare continuous variables whereas Chi-squared test was used to compare categorical variables.

## Results

During 5 years, 110 patients underwent TME with Wafi ileostomy, whereas 116 patients underwent TME with Brooke ileostomy. Propensity score matching left 99 Wafi and 99 Brooke ileostomy patients comparable for age (*p* = 0.112), gender (*p* = 1.000), and ASA score (*p* = 1.000). Table 1 summarizes demographics and perioperative outcomes in both unmatched and matched groups. BMI was significantly higher in patients undergoing Brooke ileostomy (35.7 vs. 28; *p* < 0.001). There was no difference in ASA class. The majority of patients underwent LAR in both groups. Open approach was the most common approach in Wafi ileostomy patients (80%) as compared to robotic approach in Brooke ileostomy (97%). Overall complication rates after rectal resection was higher in Wafi ileostomy patients as compared to Brooke ileostomy (17% vs. 6%; *p* = 0.015). Anastomotic leak rates did not differ statistically (11% vs. 4%; *p* = 0.104).

**Table 1:** Comparison of demographics and perioperative outcomes in unmatched and matched groups of Wafi ileostomy and Brooke ileostomy

Variables	All patients			Matched patients		
	Wafi ileostomy (*n* = 110)	Brooke ileostomy (*n* = 116)	*p* value	Wafi ileostomy (*n* = 99)	Brooke ileostomy (*n* = 99)	*p* value
Age (years)	56 (50–66)	58 (51–67)	0.395	58 (51.5–67)	57 (49.5–63.5)	0.112
Male:female ratio	72:38	74:42	0.794	62:37	63:36	1.000
BMI (kg/m^2^)	27 (25–30)	35 (28–41)	< 0.001	28 (25–31)	35.7 (28–41)	< 0.001
ASA class (*n* (%))			< 0.001			1.000
I	11 (10.0%)	0 (0%)		0 (0%)	0 (0%)	
II	81 (73.6%)	88 (75.8%)		81 (81.8%)	81 (81.8%)	
III	18 (16.3%)	24 (20.69%)		18 (18.2%)	18 (18.2%)	
IV	0 (0%)	4 (3.4%)		0 (0%)	0 (0%)	
Surgical approach (*n* (%))			< 0.001			< 0.001
Open	88 (80.0%)	6 (5.2%)		79 (79.8%)	3 (3.0%)	
Laparoscopy	11 (10.0%)	0 (0%)		1 (1.0%)	0 (0%)	
Robot	1 (0.9%)	110 (94.8%)		9 (9.1%)	96 (97.0%)	
Conversion	10 (9.1%)	0 (0%)		10 (10.1%)	0 (0%)	
Type of resection (*n* (%))			< 0.001			< 0.001
AR	2 (2.0%)	12 (10.6%)		1 (1.1%)	12 (12.5%)	
LAR	74 (73.3%)	93 (82.3%)		67 (74.3%)	77 (80.2%)	
ISR	15 (14.9%)	5 (4.4%)		13 (14.4%)	4 (4.2%)	
TaTME	1 (1.0%)	0 (0%)		1 (1.1%)	0 (0%)	
Other	9 (8.9%)	3 (2.7%)		8 (8.1%)	3 (3.1%)	
Complication rate after rectal resection (*n* (%))	17 (15.5%)	7 (6.0%)	< 0.001	17 (17.2%)	6 (6.1%)	0.015
Clavien–Dindo class (*n* (%))			< 0.001			< 0.001
0	0 (0.0%)	108 (94.7%)		0 (0%)	94 (96.9%)	
I	105 (95.5%)	0 (0.0%)		94 (94.9%)	0 (0%)	
II	5 (4.5%)	0 (0.0%)		5 (5.1%)	0 (0%)	
IIIA	0 (0%)	1 (0.87%)		0 (0%)	0 (0%)	
IIIB–IV	0 (0%)	4 (3.5%)		0 (0%)	3 (3.1%)	
V	0 (0%)	1 (0.87%)		0 (0%)	0 (0%)	
Colorectal anastomotic leak rate (*n* (%))	11 (10.0%)	5 (4.3%)	0.096	11 (11.1%)	4 (4.0%)	0.104
Mortality rate (*n* (%))	0 (0.0%)	1 (0.9%)	0.495	0 (0.0%)	0 (0.0%)	1.000

BMI, body mass index; ASA, American Society of Anesthesiologists; AR, anterior resection; LAR, low anterior resection; ISR, inter-sphincteric resection; taTME, transanal TME

Table 2 summarizes the postoperative outcomes related to ileostomy creation. Ileostomy-related overall complication rates were significantly lower in Wafi ileostomy patients (6% vs. 24%, *p* = 0.001). Five patients developed SSI and one patient had dehydration due to high ileostomy output. In Brooke ileostomy group, 22 patients developed dehydration due to high ileostomy output, 3 patients developed SSI, and one patient had postoperative small bowel obstruction requiring reoperation. Median ileostomy output did not differ between the groups (588 vs. 650; *p* = 0.145). On multivariable logistic regression, dehydration was found to be associated with increased emergency room visits and readmissions in Brooke ileostomy patients (OR = 1.24 (1.03, 3.92); *p* = 0.044).

**Table 2:** Postoperative outcomes of ileostomy creation in the Wafi and Brooke ileostomy groups

Variables	Wafi ileostomy (*n* = 99)	Brooke ileostomy (*n* = 99)	*p* value
Permanent ileostomy (*n* (%))	0 (0%)	2 (2%)	0.157
Permanent colostomy (*n* (%))	0 (0%)	1 (1%)	0.135
Ileostomy output (ml)	588 (150–1650)	650 (450–1100)	0.145
Discharged on IV fluids (*n* (%))	0 (0%)	10 (10%)	< 0.001
Rate of complications of ileostomy (*n* (%))	5 (5%)	24 (24%)	0.001
Dehydration (*n* (%))	1 (1%)	22 (22%)	< 0.001
Bowel obstruction (*n* (%))	0 (0%)	1 (1%)	0.495
SSI (*n* (%))	5 (5%)	3 (3%)	0.772
Parastomal skin irritation (*n* (%))	3 (3%)	7 (7%)	0.028
IR drainage	1 (1%)	1 (1%)	1.000
Reintervention	0 (0%)	1 (1%)	0.495
Length of hospital stay (days)	13 (11–25)	7 (5–10)	< 0.001
ER visits or readmission	1 (1%)	23 (23%)	< 0.001

IV, intravenous; SSI, surgical site infection; IR, interventional radiology; ER, emergency room

The outcomes of ileostomy reversal are summarized in Table 3. Ileostomy was not reversed in two patients with Brooke ileostomy. Median time to ileostomy reversal was significantly shorter in the Wafi ileostomy group (15 vs. 148 days; *p* < 0.001). Post-ileostomy reversal overall complication rates did not statistically differ (4% vs. 9%; *p* = 0.323). Time to ileostomy site healing was significantly shorter in Wafi ileostomy patients (6 vs. 25 days; *p* < 0.001). Medial total length of stay did not differ statistically (13 vs. 12 days; *p* = 0.244).

**Table 3:** Complications of ileostomy reversal

Complications	Wafi ileostomy (*n* = 99)	Brooke ileostomy (*n* = 99)	*p* value
Time to ileostomy reversal (days)	15 (15–25)	150 (120–240)	< 0.001
Ileostomy site healing time (days)	6 (2–30)	25 (23–35)	< 0.001
Rate of complications after ileostomy reversal (*n* (%))	4 (4%)	9 (9%)	0.323
Postoperative ileus (*n* (%))	2 (2%)	1 (1%)	0.561
Wound infection (*n* (%))	2 (2%)	6 (6%)	0.149
Electrolyte imbalance (*n* (%))	0 (0%)	2 (2%)	0.155
Total LOS (days)^a^	13 (11–25)	12 (9–17)	0.244

^a^Total LOS was defined as length of stay from index rectal cancer surgery to discharge with reversed ileostomy

## Discussion

### Context of current evidence

The main finding of this study was that Wafi ileostomy was associated with decreased rates of postoperative ileostomy-related complications, especially dehydration due to high ileostomy output as well as significantly shorter time to ileostomy reversal with fewer post-ileostomy reversal complications. In essence, although Wafi ileostomy increased LOS, it allowed to avoid ileostomy reversal procedure per se, since the reversal consisted of a tube removal at bedside during either the same hospital stay or at postoperative follow-up in outpatient clinic. Given the fact that Wafi ileostomy allowed to avoid the ileostomy reversal surgery, comparing the length of stay of the former to the first admission of the Brooke ileostomy would not reflect a fair comparison. Therefore, we compared total LOS in patients undergoing both procedures, which was defined as the length of stay from the index rectal cancer surgery to discharge with reversed ileostomy and showed no statistical difference between the procedures.

Since its first description by Brooke in 1952, ileostomy has become a standard procedure to protect distal colorectal anastomoses [5]. Although loop ileostomy does not prevent leaks, it is a commonly used procedure to prevent serious complications of anastomotic leaks, such as fecal peritonitis and sepsis [19]. Routine use of loop ileostomy has been debated over the past few decades given the rate of ostomy complications ranging from 9 to 74% [20]. Moreover, it was previously shown on large series that about 1/3 of patients do not get their ostomies reversed before death [21]. To address the downsides of Brooke ileostomies, multiple alternatives have been proposed, one of which was tube ileostomy. The idea was always to decrease ostomy-related morbidity and eliminate need for reversal. A recent meta-analysis emphasized that there was a re-emergence of interest in tube ileostomy and its anastomotic leak rates did not significantly differ from those of loop ileostomy [16]. In fact, multiple authors have described different techniques. Majority of them shared a common disadvantage, namely incomplete or inefficient diversion of fecal stream, in addition to tube malfunction due to kinking [22, 23]. Wafi ileostomy technique allows to obstruct the efferent limb of the ileostomy, thereby providing a complete diversion of fecal stream.

### Clinical and scientific implications

As reported in this study, Wafi ileostomy may provide optimal outcomes. Nonetheless, its technique needs to be standardized in minimally invasive procedures since increasingly more rectal cancer resections are performed laparoscopically and/or robotically worldwide. With regard to scientific implications, studies with larger sample size and/or of random order design are needed to confirm these findings.

### Strengths and limitations

This study compared a new ileostomy technique to a well-established common practice. One of the strengths of this study was collection of the data in a prospective fashion, which reduces the risk of recall or reporting bias. However, there are a few limitations that the authors would like to acknowledge. First limitation is the fact that the study intervention and its comparator were performed in two different institutions in different countries. This may have introduced considerable heterogeneity to the data given potential differences in perioperative care, healthcare and insurance system, and population related factors. Another limitation is that Wafi ileostomy was performed via laparotomy in the vast majority of patients. An additional limitation to this study was a relatively short follow-up.

## Conclusion

Compared to Brooke, Wafi ileostomy without need of reversal surgery and tube removal within the same hospital stay was associated with fewer complications. The diverting tube ileostomy technique using an easily removable rubber strip to defunction the colorectal anastomosis is a safe and effective method that precludes the need to create a conventional ostomy.

## Data Availability

The datasets generated during and/or analysed during the current study are available from the corresponding author on reasonable request.
